# Unveiling the profound influence of sucralose on metabolism and its role in shaping obesity trends

**DOI:** 10.3389/fnut.2024.1387646

**Published:** 2024-07-02

**Authors:** Ankul Singh S, Srishti Singh, Rukaiah Fatma Begum, Sukanya Vijayan, Chitra Vellapandian

**Affiliations:** ^1^Department of Pharmacology, Faculty of Pharmacy, Dr.M.G.R. Educational and Research Institute, Chennai, Tamil Nadu, India; ^2^Department of Pharmacology, SRM College of Pharmacy, SRM Institute of Science and Technology, Chengalpattu, Tamil Nadu, India; ^3^Department of Pharmacognosy, SRM College of Pharmacy, SRM Institute of Science and Technology, Chengalpattu, Tamil Nadu, India

**Keywords:** artificial sweeteners, digestive enzymes, obesity, sucralose, metabolic effect

## Abstract

Artificial sweeteners, prominently exemplified by sucralose, have become pervasive in contemporary diets, prompting intriguing questions about their impact on metabolism and their potential role in the unfolding trends of obesity. Covering topics from its discovery to analytical methods for detection and determination in food samples, the manuscript scrutinizes the metabolic effects of sucralose. Notably, the association between sucralose intake and obesity is examined, challenging the conventional belief of its role in weight management. The document comprehensively examines *in vivo* studies, revealing sucralose's implications on insulin resistance, gut microbiota, and metabolic syndrome, providing a nuanced comprehension of its impact on human health. Additionally, it explores sucralose's effects on glucose and lipid metabolism, blood pressure, and cardiovascular health, underscoring its possible involvement in malignancy development. The review concludes with a call for increased public awareness, education, and updated dietary guidelines to help individuals make informed choices about sweetener consumption. The future perspectives section highlights the need for longitudinal studies, exploring alternative sweeteners, and refining acceptable daily intake limits to ensure public health recommendations align with evolving regulatory guidelines. Overall, the manuscript provides a comprehensive overview of sucralose's multifaceted impact on health, urging further research and a balanced perspective on sweetener consumption.

## 1 Introduction

Artificial sweeteners have become ubiquitous in contemporary diets, serving as essential components in various food and beverage products. Among the most frequently employed artificial sweeteners in the beverage industry are aspartame, acesulfame, saccharin, sucralose, and neotame. These sweeteners play a crucial role in enhancing the palatability of diet drinks and candies, offering a sweet taste but with fewer or no caloric impact of sucrose (1). Sucralose is ~600 times sweeter than sucrose. Its high sweetness intensity means it is used in very small amounts to achieve the desired sweetness level, which can impact gut microbiota even at low concentrations (2). Aspartame is about 200 times sweeter than sucrose. It is widely used in diet beverages and food products and metabolized differently, which may influence its effects on gut microbiota and overall health (3). Saccharin is around 300–400 times sweeter than sucrose. Its use has declined due to concerns about its safety, but it is still relevant in certain food and beverage products (4). Acesulfame potassium (Ace-K) is about 200 times sweeter than sucrose. It is often used in combination with other sweeteners to enhance sweetness and stability (5).

Sucralose, marketed as Splenda, stands out due to its synthetic nature and remarkable sweetening capacity and its stability under various temperature and pH conditions, makes it suitable for use in both cooking and baking, as well as in processed foods and beverages. The sucrose (C_12_H_19_C_l3_O_8_) has a molecular weight of 397.63 g/mol with a white crystalline structure (6). Sucrose is thermally stable, water-soluble, and non-toxic in nature, making it an ideal sweetener for a wide range of culinary applications. However, due to its high caloric content, alternative sweeteners such as sucralose are often used to provide the desired sweetness without the associated calories. Artificial sweeteners serve multiple purposes of sweetening, stabilizing emulsions, and antibacterial properties, making them suitable for multipurpose additions (7, 8). The artificial sweetener market is experiencing significant growth, with global revenue projected to rise from $21.3 billion in 2021 to $28.9 billion by 2026 (9). This surge underscores the expanding use of these sweeteners across diverse industries, from food and medications to animal feed and personal care items (10). Nonetheless, the widespread consumption of artificial sweeteners, including sucralose, has raised concerns regarding potential health risks. Regulatory bodies, such as the FDA, have established acceptable daily intake (ADI) limits. For sucralose, the ADI for humans is set at 5 mg/kg body weight (11). Sucralose is an artificial sweetener commonly used in various food and beverage products. When humans consume sucralose-containing products, the compound is not significantly metabolized by the body and is largely excreted unchanged in the urine. This means that sucralose passes through the digestive system with minimal absorption and is eliminated, contributing to its low-calorie profile and making it a popular choice for those seeking to reduce sugar intake (12).

Sweeteners play a crucial role in enhancing the palatability of diet drinks and candies, offering a sweet taste without the caloric impact of sucrose. Furthermore, links between artificial sweetener consumption and an elevated risk of heart disease and type 2 diabetes have been reported, raising concerns about their long-term health effects and necessitating further research into their metabolic impacts (13). Moreover, the human body does not metabolize the vast majority of sucralose consumed; it simply passes through the GI tract unabsorbed. Consequently, sucralose is often recommended as a sugar alternative for individuals managing diabetes or those aiming to reduce overall caloric intake without sacrificing sweetness (2, 14, 15). Thus, the excessive intake of artificial sweeteners has been associated with increased appetite, weight gain, and oxidative damage (16–18). Beyond their sweetness, artificial sweeteners contribute bitterness and metallic tastes. The complexity of taste perception involves alterations in taste quality and intensity during consumption, with variations in profiles related to onset, persistence, and decay. These factors can significantly influence consumer preferences and acceptance, as the initial sweet taste may be followed by lingering bitter or metallic aftertastes (19). Understanding these dynamics is essential for food scientists and manufacturers aiming to create palatable, lower-calorie products that meet consumer expectations.

Metrics such as sweetness growth rate and sweetness potency help quantify these changes. The rate of change in sweetness intensity per unit of sweetener content was characterized as the sweetness growth rate. This metric allows for the comparison of how quickly the sweetness of a product increases with the addition of a sweetener, providing insights into its effectiveness in enhancing taste. Sweetness potency, on the other hand, was defined as the ratio of a sweetener's concentration to sucrose concentration at an equivalent sweetness intensity. This measure offers a standardized way to assess the relative sweetness of different sweeteners compared to sucrose. A higher sweetness potency indicates that a smaller amount of the sweetener is needed to achieve the same level of sweetness as sucrose, making it a valuable parameter for product formulation and optimization (20).

This review aims to explore artificial sweeteners, focusing mainly on sucralose, in today's diets and industries. It will discuss methods for testing the quality of products containing sucralose. With the rising use of artificial sweeteners, especially sucralose, the review will examine health concerns, particularly their effects on gut bacteria, blood sugar, and fat buildup. Ultimately, it seeks to offer a thorough analysis of artificial sweeteners' current status, including their use, health effects, and broader impact on individuals.

## 2 Overview of sucralose discovery and complications

Sucralose is a disaccharide composed of 4-chloro-4-deoxygalactose and 1,6-dichloro-1,6-dideoxyfructose. Inadvertently, sucralose was found in 1976 by scientists at Tate & Lyle, a British-based multinational agribusiness. The discovery was made by Leslie Hough and his team of researchers at Queen Elizabeth College, University of London (21, 22). The Food and Drug Administration (FDA) authorized the use of sucralose in April 1998 for various purposes, including as a tabletop sweetener for beverages and desserts. In 1999, it received approval to be used as a general sweetener, expanding its permitted uses beyond specific food categories (21, 23). Sucralose may make it more difficult for the body to control calorie intake, which could boost cravings for sweet, high-calorie foods. This behavior might factor in weight gain and metabolic problems. Additionally, research suggests that sucralose consumption may disrupt the body's natural mechanisms for regulating hunger and satiety, potentially leading to overeating and subsequent weight gain. Furthermore, some studies have indicated a possible link between sucralose consumption and alterations in gut microbiota, which could also contribute to metabolic disturbances and weight management challenges (24). The 12-week administration of Splenda (A small portion of sucralose as an active ingredient) has several adverse impacts. Studies have shown that prolonged exposure to Splenda can lead to a decrease in the abundance of beneficial fecal microbiota, potentially disrupting the balance of gut bacteria crucial for digestive health. Furthermore, Splenda consumption has been associated with an increase in fecal pH, which may indicate alterations in gastrointestinal function. Additionally, the expression levels of certain enzymes, including P-glycoprotein (P-gp), cytochrome P450 3A4 (CYP3A4), and cytochrome P450 2D1 (CYP2D1), have been observed to elevate with Splenda usage. These enzymes are known to play roles in drug metabolism, particularly in reducing the bioavailability of medications taken orally. Elevated expression of these enzymes may interfere with the effectiveness of certain medications, potentially compromising therapeutic outcomes and necessitating adjustments in dosing or medication selection (25). The adverse effects associated with sucralose consumption have been detailed in Table 1 for *in vivo* studies, while Table 2 specifically focuses on the adverse effects observed along with limitations in various *in vivo* models.

**Table 1:** Adverse health effects linked to sucralose consumption.

**Food sample (sucralose rich)**	**Adverse health effects**	**References**
Juices, teas, diary based drinks, beers	Headache, mood swings, depression, compromised memory, brain tumors, leukemia, seizures	(26)
Carbonated cola drinks, fruit drinks, Tea drinks	Obesity and chronic inflammation	(27)
Solid sweets, soft drinks, sweetener pills	Mental disorders, type 2 diabetes	(28)
Soft drinks, low-calorie foods	Impaired kidney and liver function, obesity, cancer	(29)
Cola drinks, jams, soft drinks	Hypertension, obesity, metabolic syndrome, diabetes, cardiovascular diseases	(30)
Wine, soya sauce, pickles	Reduce Hb levels, RBC and WBC count, elevation in liver and kidney enzymes	(31)
Chewing gums, teas, cocoa mixes	Elevated liver enzymes, cholesterol	(32)
Soft drinks, desserts, jellies, frozen puddings	Headache, hepatotoxicity	(33)
Wine beers, candies, canned mangoes, canned peaches, cakes	Headache, dizziness, gastrointestinal issues	(34)
Desserts, ice cream, soft drinks, candies	Edema, hypertension, electronic imbalance	(35)
Baked foods, cold and hot beverages	Type 2 diabetes, obesity	(1)

**Table 2:** *In vivo* models assessing the adverse effects of sucralose.

**Methodology/type of study**	**Level of evidence**	**Population/animal population**	**Adverse effects**	**Objectives and findings**	**Limitation**	**References**
*In vivo* preclinical model	Weaker	Rat models, *n* = 50	Altered gut microbiota composition linked to sucralose consumption.	Examining sucralose effect on gut microbiome of mice (6-month study). Assessment of inflammation markers to define the effects of sucralose consumption. Sucralose altered the gut microbiome and associated metabolic profiles, which may contribute to inflammatory response in the mouse liver.	Lack of direct human evidence, limited generalizability.	(36, 37)
Meta-analysis	Strongest	Various clinical studies	Reported association between sucralose and increased risk of obesity.	Assessment of the association between obesity and consumption of sugar and artificially sweetened beverages. A significant association between sugar and artificially sweetened soda consumption and obesity is noted.	Heterogeneity among included studies, potential publication bias.	(38, 39)
Double-blinded randomized controlled trial (RCT)	Stronger	Healthy young adults, *n* = 137	Increased insulin resistance compared to control group.	Investigation of acute or chronic sucralose ingestion behavior on insulin or glucose alterations in healthy young individuals that daily consume 48 or 96 mg sucralose for 10 weeks, a sucralose amount equivalent to one or two diet sodas, respectively. Chronic consumption of sucralose can affect insulin and glucose responses in non-insulin-resistant healthy young adults with normal body mass index which still needs further research to confirm.	Short duration, limited diversity in the participant group.	(40)
Cohort prospective study	Strong	Mother-singleton child dyads population, *n* = 918	Childhood risk of overweight/obesity	Intake of artificial sweetener and sugar-sweetened beverages during pregnancy in relation to offspring growth through age 7 years among high-risk children born to women with gestational diabetes. The study showed positive associations between intrauterine exposure to ASBs and birth size and risk of overweight/obesity at 7 years.	Limited long-term data, potential recall bias.	(41)

In level of evidence: meta-analysis and systematic review are strongest; randomized controlled trials are stronger; cohort studies are strong; case-control studies are medium; cross-sectional studies are weak; animal trials and *in vitro* studies are weaker; case reports, opinion papers and letters are weakest.

## 3 Determination of sucralose in a variety of food samples (analytical)

Analytical methods are essential for ensuring the safety and quality of food products. These methods can be used to monitor compliance with regulations, detect adulteration, and conduct research on sweeteners by identifying their presence and concentration in various products, assessing their stability under different conditions, and evaluating their metabolic and health impacts (42). Analytical methods, including high-performance liquid chromatography, flow-injection analysis, ion chromatography, thin-layer chromatography, capillary electrophoresis, gas chromatography, and electroanalysis, are applicable for the individual or simultaneous analysis of sweeteners within mixtures (43, 44). High-performance LC and capillary electrophoresis, along with anion-exchange chromatography and reverse-phase HPLC, offer effective separation and quantification methods for sucralose (34). The analytical technique used will be determined by the sweetener being investigated, the concentration of the sweetener in the sample, and the required degree of accuracy and precision.

In addition to the analytical methods mentioned above, several spectroscopic methods can be used to determine sucralose in food samples, including IR and NMR. Infrared (IR) spectroscopy can identify functional groups and molecular structures by measuring the absorption of infrared light at different wavelengths. Nuclear Magnetic Resonance (NMR) spectroscopy provides detailed information about the molecular structure and dynamics of sucralose by observing the behavior of nuclei in a magnetic field. These spectroscopic techniques complement other analytical methods by offering precise and non-destructive ways to analyze the presence and concentration of sucralose in various food matrices (45).

High-performance LC (HPLC) is a universal technique with a broad range of separation columns and detectors, including UV-visible, IR, Mass spectrometry, light scattering, and conductivity detector, making it ideal for sweetener separation (43). The basic concept of HPLC separation of sucralose in analytical samples is that analytes have distinct affinities to the stationary phase column and the mobile phase. During the HPLC process, the sample mixture is injected into the column, where each component interacts differently with the stationary phase. Components with higher affinity for the stationary phase move more slowly, while those with greater affinity for the mobile phase move faster. This differential migration results in the separation of sucralose from other components in the sample, allowing for its precise detection and quantification (46). UHPLC with a diode array detector enables fast separation and has been used to analyze sucralose in analytical samples (47). Capillary electrophoresis is an effective food analysis technology due to its excellent resolution, low solvent use, and ease of automation, making it a viable alternative to HPLC (48).

Sucralose, lacking a chromophore, does not absorb UV light. However, it can undergo photochemical oxidation to form a UV-active carbonyl compound that absorbs light at 270 nm. When exposed to UV light for 1 h at acidic or neutral pH, a new absorption band appears at 270 nm. The optimal pH for this reaction was identified by testing different alkaline pH levels (49). Simple and rapid methods for quantifying sucralose in food products were established, employing anion-exchange chromatography (AEC) and reverse-phase HPLC. The process involved injecting sucralose concentrate into AEC, followed by liquid extraction with water or methanol and a subsequent clean-up using a solid-phase extraction (SPE) cartridge. The results of the final determination revealed recovery rates of sucralose from foods ranging from 80.6 to 102.0% (50).

## 4 Metabolic effects of consuming sucralose compared to other artificial and natural sugars

Sucralose, although not fully absorbed in the digestive tract, can still influence the gut microbiota. The interaction of sucralose with gut bacteria leads to the production of metabolites that can affect the liver's function, which is crucial for detoxification. These downstream-released metabolites have the potential to dysregulate host metabolism by interfering with normal metabolic processes and contributing to metabolic disorders (36). Sucralose, an artificial sweetener, has been shown to alter the homeostasis of the gut microbiota in mice, potentially leading to liver tissue inflammation. Research indicates that sucralose consumption significantly impacts various biological processes and functions in the mouse liver, including metabolic pathways and inflammatory responses. These changes may contribute to adverse health effects, highlighting the need for further investigation into the long-term implications of sucralose consumption (51). Nonetheless, it's improbable that sweeteners will enhance glucose regulation due to their potential effects on the body, such as altering intestinal glucose transport and uptake, contributing to insulin resistance, and reducing insulin secretion capability (52). According to a study, sucralose does not undergo active transport through the blood-brain barrier, the placental barrier, or the mammary gland. This means that sucralose, once ingested, does not readily pass into the brain, the developing fetus, or breast milk. As a result, sucralose is less likely to have direct effects on the central nervous system, fetal development, or nursing infants. This limited transport reduces potential risks associated with sucralose consumption in these sensitive areas, contributing to its safety profile as a non-caloric sweetener (45). About 2% of orally ingested sucralose is metabolized into components of negligible toxicity and eliminated through urine. The remaining sucralose is excreted unchanged in the feces. Sucralose does not exhibit any significant affinity for binding with blood proteins or other proteins, which contributes to its minimal impact on metabolic processes and its safety profile as a non-nutritive sweetener (24, 53).

Natural sugars like fructose and lactose are digested slower than added sugar (artificial sweeteners like Sucrose, fructose etc.,) ensuring a more stable metabolism. Plant-based sources of natural sugars, such as fruits and vegetables, offer a nutritious alternative to refined sugar and may even provide protective benefits against certain diseases. These sources are rich in vitamins, minerals, fiber, and antioxidants, offering more than just empty calories and contributing to overall health (54). Date plant fructose lowers glycemia after ingestion because it is delivered in modest amounts into the bloodstream. This controlled release helps maintain lower blood sugar levels in both healthy individuals and those with hyperglycemia (55). Sucrose, glucose, fructose, galactose, mannose, and arabinose are all found in sweet sorghum juice. Tannins and polycosanols in sweet sorghum have been linked to weight loss and improved heart function (56, 57). Honey, a natural sweetener, possesses antioxidant properties that can potentially mitigate oxidative stress disorders such as aging, cardiovascular diseases, diabetes, and renal failure. Its bioactive compounds, including flavonoids and phenolic acids, help neutralize free radicals in the body, thereby reducing oxidative damage and inflammation associated with these conditions (58). The consumption of natural sugars provides more health benefits than artificial sweeteners. Both natural sugars and artificial sweeteners have their respective advantages and considerations. Nonetheless, metabolic effects of various artificial sweeteners have been discussed in Table 3, respectively. While natural sweeteners are often considered healthier alternatives to refined sugars, they are not without their disadvantages. It's important to note that the impact of natural sweeteners can vary based on factors such as individual health conditions, dietary requirements, and consumption patterns. While the examined natural sweeteners may offer numerous health-related benefits, notable disadvantages are evident in their extraction processes, which directly impact their ultimate physicochemical characteristics. Various innovative methods are being explored to enhance both the overall production yield and quality (66). To address these challenges, researchers are developing advanced extraction techniques that aim to improve efficiency and sustainability. These methods include enzymatic treatment, fermentation, and green chemistry approaches, which minimize the use of harmful solvents and reduce waste. By optimizing these processes, it is possible to enhance the yield and quality of natural sweeteners, making them more accessible and environmentally friendly. It is crucial for individuals to make informed choices based on their health needs, dietary preferences, and overall wellbeing. The emphasis is on maintaining a balanced perspective, recognizing that moderation and mindful consumption play key roles in achieving optimal metabolic health.

**Table 3:** A comparative analysis of metabolic effects and safety profiles of sucralose and other sweeteners.

**Sweetener**	**Metabolic effects**	**Safety considerations**	**Regulatory status**	**Referencess**
Sucralose	Impact on blood glucose, gastrointestinal disorder symptoms, affect insulin and glucose responses in non-insulin resistant healthy young adults	Generally recognized as safe in acceptable daily intake limits	Approved by FDA, EFSA, and other regulatory bodies.	(40, 59, 60)
Sucrose	May impact blood glucose, caloric content	Nutrient content, potential for overconsumption leading to obesity and related issues	Regulated as food ingredients, with specifications on use.	(61)
Fructose	Natural sugar is found in fruits and honey. Insulin resistance and increased fat accumulation	Nutrient content, potential for overconsumption leading to obesity and related issues	Regulated as food ingredients, with specifications on use.	(62)
Aspartame	Low-calorie artificial sweetener.	Safety concerns may include potential links to health conditions (e.g., headaches)	Approved by FDA, EFSA, and other regulatory bodies with specified ADI limits.	(63)
Stevia	Managing diabetes or reducing calorie intake.	Joint FAO/WHO Expert Committee on Food Additives (JECFA) and the U.S. Food and Drug Administration (FDA) have established acceptable daily intake (ADI) levels.	Approved by FDA, EFSA, and other regulatory bodies. Specific regulations may vary by country.	(64)
Saccharin	Negligible impact on blood glucose levels.	generally recognized as safe (GRAS)	Approved by FDA, EFSA, and other regulatory bodies. Specific regulations may vary by country.	(65)

## 5 Sucralose consumption and its association with obesity

Sucralose consumption has been linked to weight gain rather than weight loss, and it has been shown to enhance hunger and food intake. Additionally, studies have indicated that the blood uric acid levels of those consuming more sugar-sweetened beverages were greater, suggesting a potential metabolic impact associated with increased consumption of sucralose-containing products (67, 68). Moreover, research has shown that uric acid can trigger renal inflammation through the NF-κB signaling pathway in mice experiencing hyperuricemia. Furthermore, artificial sweeteners like sucralose and fructose have been found to elevate NF-κB activity. This elevation is attributed to a reduction in the transrepression activity of peroxisome proliferator-activated receptor-α (PPAR-α), a key regulator of inflammation and metabolic processes. Thus, the consumption of sucralose and fructose may contribute to the activation of inflammatory pathways, potentially exacerbating renal inflammation and associated complications (69, 70).

**Figure 1:**
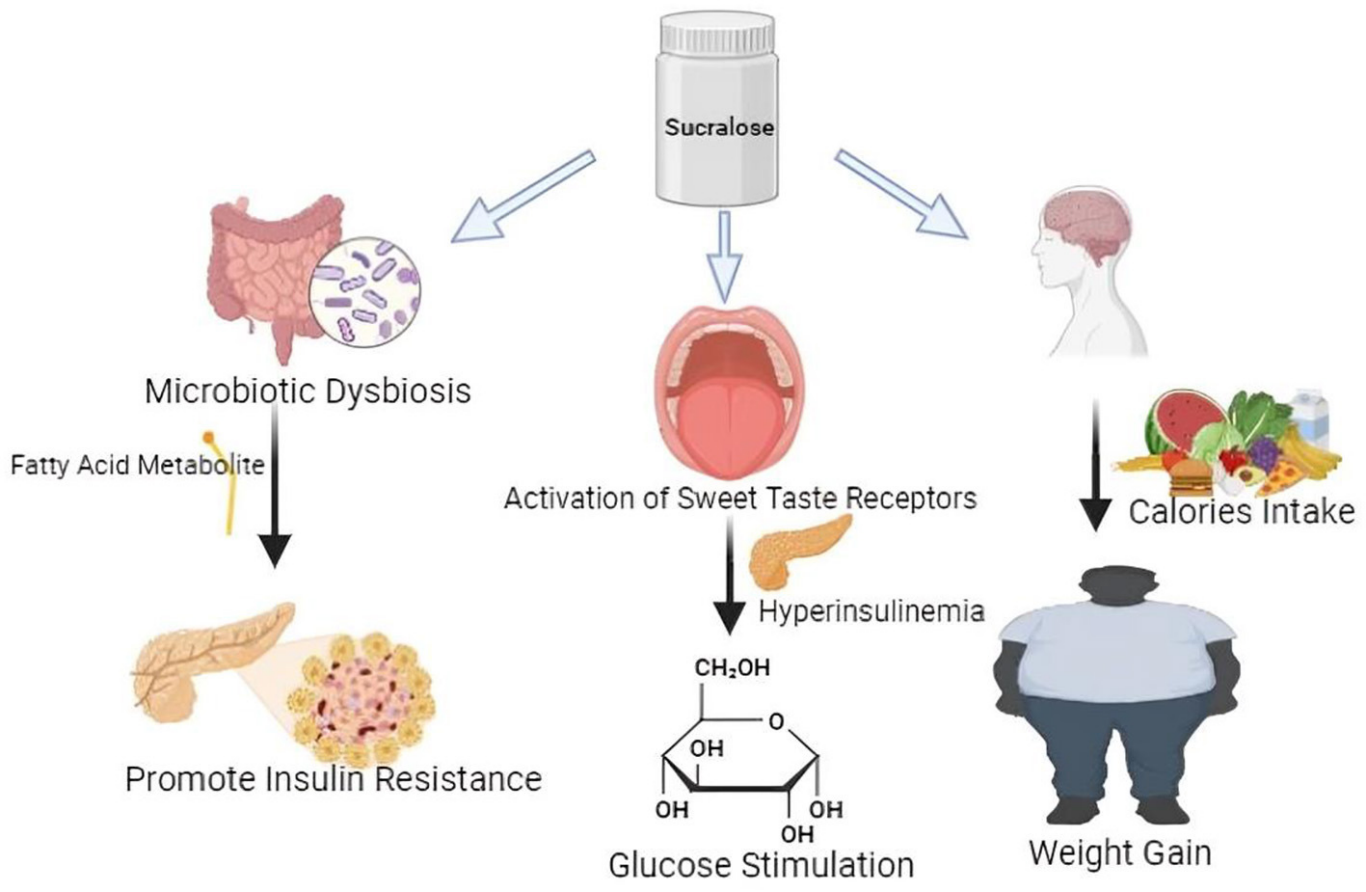
Effects of sucralose on insulin resistance and adiposity.

In the context of lipogenesis, the pivotal enzyme is fatty acid synthase (FAS), whereas intracellular triglyceride breakdown involves adipocyte triglyceride lipase (ATGL) and hormone-sensitive lipase (HSL). The modulation of these enzymes is significant in understanding the metabolic effects of sucralose. Studies have demonstrated that sucralose intake can influence the activity and expression of these enzymes, thereby impacting lipid metabolism. For instance, research on mice fed a high-fat diet has shown that sucralose supplementation reduces glucose intolerance and improves gluconeogenesis, indicating its potential role in ameliorating metabolic dysregulation associated with obesity and insulin resistance (71). The Pathway between Sucralose and elevated uric acid levels, fat, triglycerides, and insulin may be mediated by metabolic syndrome, hypertension, and obesity as illustrated in Figure 1, respectively. The average blood pressure variations are predicted by uric acid. Although obesity likely causes this correlation, uric acid levels directly correlate with body mass index (BMI), suggesting a potential independent role of uric acid in blood pressure regulation. Elevated uric acid levels have been associated with hypertension, a condition commonly linked to obesity. This correlation underscores the intricate interplay between metabolic factors such as uric acid, BMI, and blood pressure regulation, highlighting the need for comprehensive approaches to managing cardiovascular health (72–74). Sucralose upregulates SGLT-1 expression which is independent of the action of GLUT2, and improves intestinal glucose absorption, ultimately contributing to alterations in glucose homeostasis and potentially influencing metabolic outcomes such as insulin sensitivity and energy metabolism (75, 76). Sucralose increases the abundance of T1R2 and T1R3 taste receptors in the tongue and small intestinal epithelium, and they function as luminal sugar sensors to control SGLT1 expression in response to dietary sugars, ultimately modulating glucose absorption and potentially influencing metabolic processes such as glycemic control and energy homeostasis (77). This suggests that artificial sweetener-containing diets can contribute to weight gain and obesity by disrupting innate physiological and homeostatic mechanisms. The upregulation of T1R2 and T1R3 receptors by sucralose may lead to heightened sensitivity to sweetness, potentially altering taste perception and preference for sweet foods. Additionally, the dysregulation of SGLT1 expression, a crucial transporter involved in glucose absorption in the intestine, could disrupt glucose homeostasis and contribute to metabolic dysfunction. Thus, while artificial sweeteners offer a low-calorie alternative to sucrose, their impact on taste receptor expression and glucose handling mechanisms raises concerns about their potential role in promoting weight gain and obesity (78, 79).

## 6 Sucralose consumption and maternal exposure

The consumption of sucralose during pregnancy has been associated with adverse health outcomes in children, including increased obesity, body weight, insulin resistance, and microbiota dysbiosis. Studies have shown that maternal exposure to sucralose can have long-lasting effects on offspring's health, potentially predisposing them to metabolic disorders later in life. These findings underscore the importance of understanding the impact of artificial sweeteners on maternal and fetal health and highlight the need for further research into the potential risks associated with their use during pregnancy (80, 81). Another study by Halasa et al. (82) examined the levels of sucralose in amniotic fluid and umbilical cord of pregnant women and observed that those concentrations were 30.6 ng/mL. It did not trace back in cord blood samples indicating its high presence in amniotic fluid and maternal intake of sweetener during pregnancy is associated with preterm birth and higher infant weight gain in epidemiologic studies (82). Various mechanisms proposed for sweeteners like sucralose include intestinal glucose absorption, alterations in intestinal microbiota, induction of oxidative stress and a dysregulation of appetite and reward responses (83). Maternal consumption of diet sodas containing sucralose results in the transfer of this sweetener into breast milk, which may affect children who consume the milk (84). Thus, sucralose has been studied to exert its profound influence on metabolic consequences. However, a plethora of evidence needs to be ascertained to re-examine its health effects by refining methodological approaches.

## 7 The effects of sucralose on metabolic health and *in-vivo* investigation

**Figure 2:**
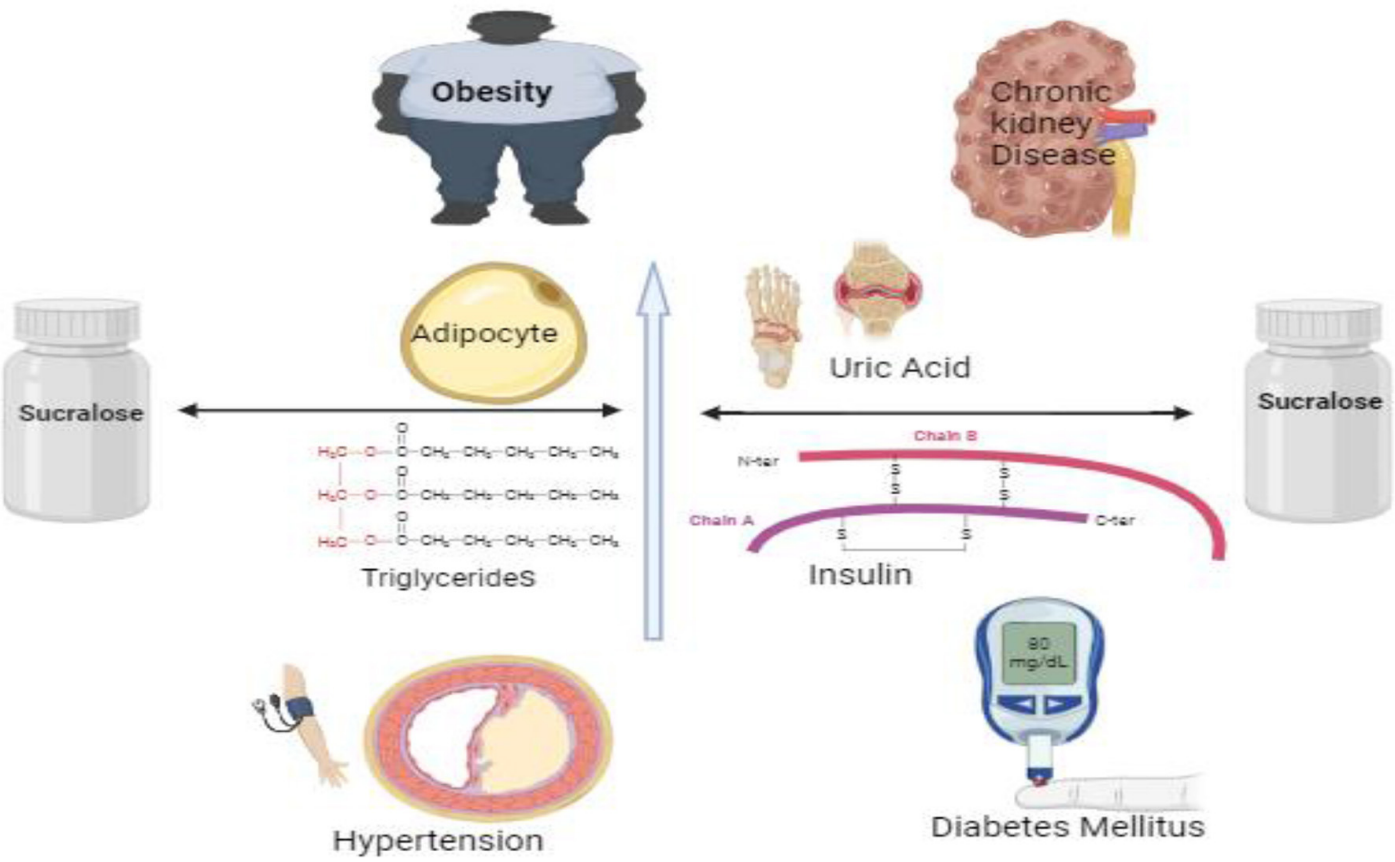
Multiple effects of sucralose in various diseases.

The ability of Sucralose to activate sweet taste receptors within mouse pancreatic beta cells highlights its multifaceted effects on metabolic regulation, suggesting a complex interplay between sweet taste perception and endocrine function. Sucralose influences insulin secretion by activating the TAS1R2/TAS1R3 taste receptor complex (85). Studies have indicated that sucralose exerts an influence on gene expression within hypothalamic cells, particularly impacting the sweet taste receptor T1R2. This modulation raises intriguing possibilities regarding the regulation of appetite, suggesting that sucralose may alter the perception of extracellular glucose levels within the brain, thereby influencing hunger and satiety signaling pathways. Notably, sucralose has been deemed safe for consumption across various demographic groups, including pregnant women, children, and individuals with medical conditions. This underscores its widespread applicability as a sugar substitute in food and beverage products (24, 86). Experimental studies utilizing isolated mouse pancreatic islets have consistently demonstrated that sucralose enhances insulin release in the presence of glucose. However, it's important to note that without absorption, sucralose has limited interaction with pancreatic beta cells, indicating that its metabolic effects are primarily localized within the **gastrointestinal tract** (Figure 2). Despite its potential to stimulate insulin secretion, the direct impact of sucralose on pancreatic function may be contingent upon its absorption and subsequent interaction with beta cells (87, 88). A comprehensive study revealed significant alterations in adipocyte physiology following a 4-month regimen of sucralose administration. Results demonstrated an increase in adipocyte size and heightened expression of leptin, a hormone associated with adiposity. Conversely, levels of adiponectin, a hormone with anti-inflammatory and insulin-sensitizing properties, were observed to decrease, alongside reductions in uncoupling protein levels. These findings underscore the potential impact of sucralose on adipose tissue dynamics and metabolic signaling pathways (23, 89). In mice, sucralose promotes the release of two important hormones, glucagon-like peptide-1 (GLP-1) and glucagon-like peptide-2 (GLP-2), from enteroendocrine L cells (90, 91). Studies have demonstrated that administering sucralose to rats leads to an upregulation in the expression of cytochrome P-450 isozymes and the efflux transporter P-glycoprotein. These enzymes are integral components of the pre-systemic detoxification system responsible for first-pass drug metabolism. The observed increase in CYP isozymes and P-gp expression suggests a potential modulation of drug metabolism pathways in response to sucralose consumption. Understanding these alterations is crucial for assessing the implications of sucralose on drug efficacy and toxicity profiles (92, 93). In studies conducted on mice, sucrose was found to disrupt the DNA integrity within the gastrointestinal tract (GIT). Additionally, research involving rats revealed that sucrose increased glucose absorption, primarily facilitated by the diffusive apical GLUT2 pathway, and enhanced SGLT1 expression both being independent of each other presence (75). These findings underscore the potential impact of sucrose on both genetic integrity within the GIT and glucose absorption mechanisms, highlighting the importance of further investigation into the physiological effects of sucrose consumption (94).

Sucralose stays in the bloodstream for longer than 18 h after consumption, and consumption by mothers during pregnancy and lactation is thought to have long-term consequences that increase the risk of metabolic disorders (14, 95). The consumption of sucralose leads to metabolic effects by altering enzymes and proteins, thereby impacting overall health (Table 4). Investigations into the metabolic effects of sucralose have revealed its potential to modulate the body's response to an oral glucose load, impacting glycemic, insulin, and incretin responses. Research suggests that sucralose consumption may influence glucose metabolism by altering glycemic control, insulin secretion, and the release of incretin hormones. These findings underscore the need for further exploration into the intricate interplay between sucralose intake and glucose homeostasis, shedding light on its potential implications for metabolic health (102, 103). Research indicates that introducing sucralose into the diet of male Swiss mice from prenatal stages and continuing throughout their lifespan is associated with the development of hematopoietic neoplasms. These findings underscore the potential long-term impact of sucralose consumption on hematopoietic health in experimental models. Understanding the mechanisms underlying this association is crucial for assessing the safety and potential risks of sucralose consumption across different life stages (104).

**Table 4:** Metabolic effect of sucralose on enzymes.

**S.No**.	**Enzymes/proteins**	**Metabolic effect after consuming sucralose**	**References**
1.	Amylase	Rapidly inhibits glucose metabolism, resulting in long-term brain dysfunction.	(96)
2.	Proteolytic enzyme/protease	Deconjugated bilirubin-mediated inflammation, damaged gut barrier, and impaired inactivation of digesting protease	(97, 98)
3.	Pancreatic polypeptide	Increases the levels of insulin in the blood.	(99)
4.	Cytochrome P-450	The expression of cytochrome P450 and P-glycoprotein in the gut was enhanced. Because these two transporters are involved in drug detoxification, the body may perceive sucralose as a poison that must be removed. Increased expression of the CYP enzyme and P-gp in the gut.	(24)
5.	GLP-1 & GLP-2	Reduces glucagon secretion by the liver	(100)
6.	Lipase	Transesterification reaction under enzymatic conditions. Unreactive under biocatalytic conditions using lipase enzyme	(101)

## 8 Effect of sucralose on gut microbiota

**Figure 3:**
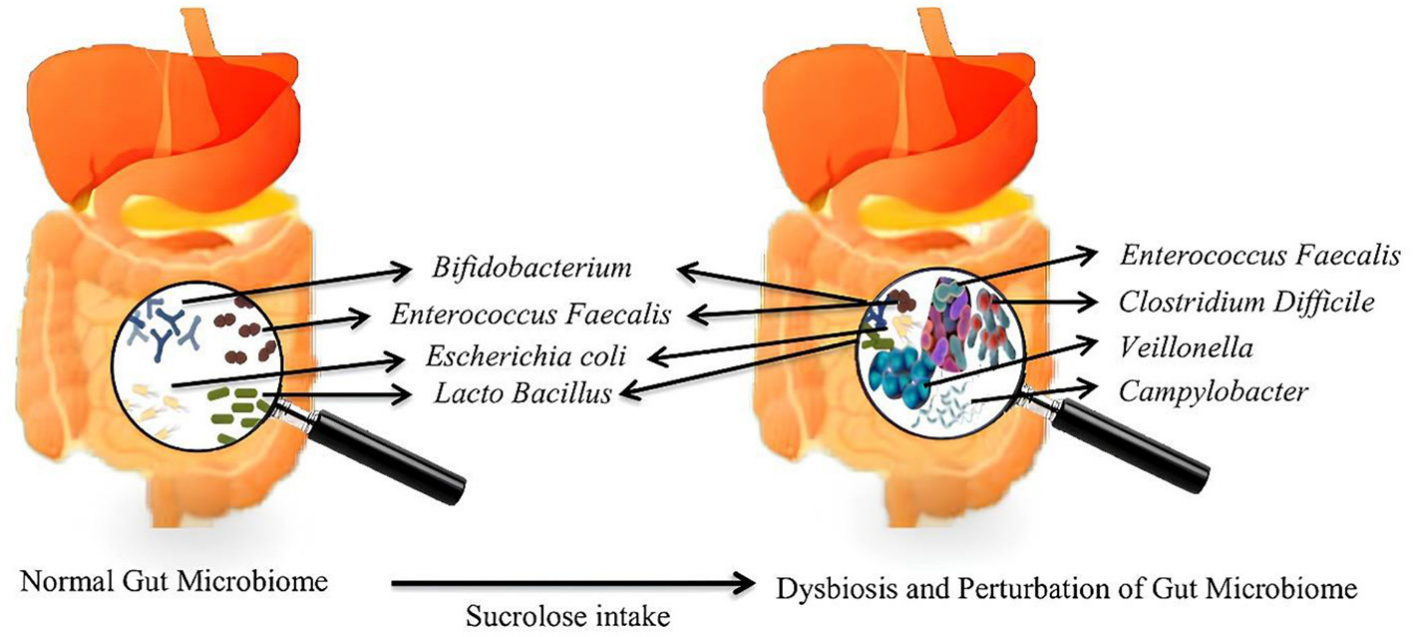
Impact of sucralose on gut microbiota composition and health outcomes.

The microbiota is the aggregate name for the trillions of symbiotic bacteria present inside humans, most of which are located in the GIT, mainly in the **rumen** (105). People and animals have different gut microbiota profiles and various microorganisms, most notably bacteria, archaea, yeasts, and viruses. Dysbiosis is a disrupted balance, decreased microbial diversity, and increased pro-inflammatory species (106). The intake of sucralose leads to a disruption in the gut microbiota, notably in the overall counts of both anaerobic and aerobic bacteria, resulting in a marked reduction in beneficial anaerobic bacteria such as Bifidobacteria, Lactobacilli, and Bacteroides (25, 107). Sucralose led to an increase in the population of Firmicutes while indicating a declining pattern in Bacteroidetes, along with reduced alpha diversity (108). Sucralose has been identified for its deleterious effect on human gut microbiota by a decline in butyrate-producing bacteria such as *Roseburia* and *Faecalibacterium prausnitzii* and increased bacterial species positively correlated with intestinal inflammation and fibrosis (i.e.,: *Enterococcus, Veillonella* and *Mucispirillum schaedleri*) (Figure 3) (109). High sucralose concentration has also shown depression-like behavior in animals (110), and the disruption of normal gut microbiota and metabolic profiles in lipid and cholesterol homeostasis of the liver (111). Sucralose thus promoted intra-and inter-genus spread between bacteria of antibiotic resistance genes in a dose-dependent manner (112). Moreover, non-caloric artificial sweeteners have been shown to induce oxidative stress and boost the plasmid-mediated conjugative transfer of antibiotic-resistance genes among the gut microbiota and a human pathogen (113). Nonetheless, low-dose sucralose administration has been shown to affect the intestinal barrier function evidenced by distinct lymphocyte aggregation in the ileum and colon while not changing the body weight of mice (114). Clinically, Sucralose consumption increases serum insulin and induces gut dysbiosis associated with altered insulin and glucose levels (115). Studies have demonstrated that exposure to sucralose in mothers changes the gut microbiota of their offspring at the time of weaning and increases the likelihood of the offspring developing obesity, non-alcoholic fatty liver disease, and metabolic syndrome later in life indicating the precautious usage of sucralose during pregnancy (116).

## 9 Impact of sucralose on body metabolism and malignancies

### 9.1 Glucose metabolism

Sucralose consumption elevated insulin levels and decreased insulin sensitivity, indicating a possible effect on glucose metabolism. Consuming sucralose has been associated with elevated insulin levels and reduced insulin sensitivity (117). Insulin resistance, characterized by elevated insulin levels and diminished cellular responsiveness to insulin, is a pivotal component in various metabolic disorders. This condition reflects an inadequate reaction of the body's cells to insulin signals, potentially culminating in persistent hyperglycemia. Disruptions in glucose metabolism associated with insulin resistance not only predispose individuals to diabetes but also exacerbate the risk of obesity. The dysregulated interplay between insulin and cellular receptors can instigate a cascade of metabolic dysfunctions, contributing to the pathogenesis of these prevalent metabolic disorders (118, 119). The utilization of sucralose has been associated with modifications in the gut microbiota composition, potentially influencing glucose homeostasis and contributing to reduced glucose tolerance and insulin resistance. Reduced glucose tolerance, characterized by impaired sugar processing and elevated blood sugar levels, may stem from disturbances in glucose homeostasis, including altered gut microbiota profiles. These alterations in microbial communities within the gastrointestinal tract have been linked to metabolic dysregulation, raising concerns about the impact of sucralose consumption on glucose metabolism and insulin sensitivity (120–122).

### 9.2 Lipid metabolism

Sucralose has been implicated in altering lipid metabolism, potentially leading to adverse effects such as increased fat storage and reduced insulin sensitivity, both of which may contribute to dyslipidemia. Studies have suggested that sucralose consumption can promote lipid accumulation, leading to elevated levels of circulating fats. Furthermore, evidence indicates that sucralose intake may impair insulin sensitivity, a key factor in regulating lipid metabolism, thus exacerbating dyslipidemia. These findings underscore the need for further investigation into the potential impact of sucralose on lipid homeostasis and its implications for metabolic health (123). Sucralose consumption has been linked to alterations in the composition of gut microbiota and an increase in the expression of intestinal P-glycoprotein and cytochrome P-450 enzymes. These enzymes play crucial roles in drug and toxin metabolism within the body. The observed changes suggest that sucralose intake may influence the body's ability to metabolize pharmaceutical drugs and environmental toxins, potentially affecting drug efficacy and toxicity levels. Understanding the impact of sucralose on these metabolic pathways is important for assessing its overall safety and potential health implications (25).

### 9.3 Blood pressure and cardiovascular disease

Changes in insulin sensitivity and glucose metabolism exert significant effects on cardiovascular health, influencing factors such as blood pressure regulation. These alterations, intricately linked with metabolic syndrome and diabetes, play a pivotal role in the pathogenesis of cardiovascular diseases. Understanding the interplay between insulin sensitivity, glucose metabolism, and cardiovascular function is crucial for unraveling the complex mechanisms underlying cardiometabolic disorders and developing targeted therapeutic interventions (120). Studies examining the health effects of sucralose have revealed notable associations between the consumption of beverages containing this artificial sweetener and intermediate markers of cardiovascular disease (CVD), such as myocardial infarction, acute coronary syndrome, angioplasty, angina pectoris, stroke, and transient ischemic attack. Specifically, there is evidence suggesting a modest increase in the unfavorable total cholesterol to HDL cholesterol ratio, as well as adverse changes in lipid profiles and leptin levels. Additionally, consumption of sucralose-sweetened beverages has been linked to an increased risk of hypertension (124–126).

### 9.4 Malignancies

A study conducted by Schiffman et al. in 2023 demonstrated that sucralose-6-acetate had a significant effect on gene expression related to inflammation, oxidative stress, and cancer. Notably, there was a marked increase in the expression of the metallothionein 1 G gene (MT1G). These findings underscore the potential implications of sucralose-6-acetate exposure on molecular pathways associated with health outcomes, particularly in the context of inflammation, oxidative stress, and carcinogenesis (34). The comprehensive assessment, utilizing rigorous categorization and evaluation based on Key Characteristics of Carcinogens (KCCs), is in concordance with conclusions drawn by reputable regulatory bodies, substantiating the safety of sucralose within its designated applications and dismissing apprehensions regarding mutagenicity and carcinogenicity. This meticulous analysis underscores the reliability of findings and bolsters the confidence in the safety profile of sucralose, aligning with the consensus of authoritative scientific bodies (127). This conclusion aligns with findings from a recent comprehensive review, which strongly affirms that sucralose does not present a carcinogenic risk. The review, characterized by its thoroughness and rigor, provides robust evidence supporting the safety of sucralose consumption in relation to carcinogenicity. These findings offer reassurance regarding the safety profile of sucralose and underscore its suitability as a non-carcinogenic sweetener option for various food and beverage products (32, 128). The divergent outcomes highlight the necessity for ongoing investigation into the multifaceted impact of sucralose on human health, facilitating a comprehensive and impartial examination of its potential ramifications. It is imperative to acknowledge the variability in responses to sucralose among individuals, considering factors such as dietary habits, genetic predispositions, and gut microbiota composition, which may significantly influence reactions to sweeteners. Continued research efforts are essential for a nuanced understanding of sucralose's effects and their implications for public health.

## 10 Strength and limitations

The overall study primarily concentrates on the risks associated with sucralose consumption, yet to achieve a comprehensive understanding, a thorough risk-benefit analysis is essential. In the analytical methods for sucralose determination in food samples, the study acknowledges the need to add potential impact of variations in sample preparation, detection methods, and instrument sensitivity on result accuracy and reliability. Therefore, the standardization and validation of these analytical techniques are deemed critical. While certain sections of the study address the long-term effects of sucralose consumption, there is an inconsistent emphasis on the duration and frequency of exposure. Notably, overall study does not extensively explore the regulatory frameworks governing sucralose use and their potential influence on public health. Understanding the context of permissible levels and regulatory oversight is crucial in this regard. The adverse health effects outlined in Table 1 are linked to sucralose-rich food samples; however, the study may not adequately consider variations in individual responses to sucralose. Factors such as genetics, pre-existing health conditions, or other dietary habits can contribute to diverse reactions among individuals.

## 11 Future perspectives

The study on sucralose consumption and its potential health implications lays the foundation for several future research directions. Longitudinal studies are imperative to unveil the prolonged effects of sucralose exposure, especially given emerging evidence of potential adverse health outcomes. Investigating the interplay between sucralose and other artificial sweeteners, along with their collective impact on metabolic health, promises a more comprehensive understanding. Furthermore, it is essential for targeted interventions to delve into in-depth mechanisms behind sucralose-induced alterations in gut microbiota and their subsequent impact on metabolic pathways. The study underscores the need for exploring sucralose's effects on specific populations, including pregnant women, children, and individuals with underlying health conditions, addressing potential developmental impacts and links to obesity and metabolic disorders in offspring. The study emphasizes the importance of exploring alternative sweeteners, both natural and artificial, to develop healthier substitutes with minimal adverse health effects. Lastly, continuous research is crucial for refining acceptable daily intake limits and ensuring that public health recommendations remain up-to-date, aligning with evolving regulatory guidelines. In summary, future investigations should delve into the intricate interactions between sucralose and human health. Given the accumulating evidence of these multiple adverse effects, healthcare providers must be aware of dietary guidelines and inform consumers about the potential health risks associated with the use of sucralose. Encourage healthcare professionals to monitor and assess patients who report adverse effects related to sucralose consumption. This will contribute to better understanding individual variations in responses. Policymakers can support public awareness campaigns to inform the general population about the healthier choices and promotes transparency in the food industry. This approach aims to empower individuals to make informed choices and encourages ongoing research and regulatory diligence in ensuring public health.

## 12 Conclusion

The extensive body of research indicates that sucralose poses a substantial health risk. Its consumption has been linked to various adverse effects, including an increased risk of metabolic syndrome, type 2 diabetes, hypertension, obesity, lipid metabolism dysregulation, hepatic inflammation, and cardiovascular disease. Additionally, the detrimental effects of sucralose extend beyond metabolic and skin-related issues. Its usage has been associated with increased fecal pH, a reduction in beneficial fecal microflora, and heightened expression levels of certain enzymes (P-gp, CYP2D1, and CYP3A4) that limit the bioavailability of orally administered drugs. Perhaps most concerning is the chemical instability of sucralose, which can lead to the release of chlorinated aromatic polycyclic hydrocarbons (CI-PAHs) in the body. These toxic compounds have been found to accumulate and are potentially carcinogenic, with links to various types of cancers in humans. It is advisable to adhere to the European Union Scientific Committee's recommendation of a daily intake of no more than 15 mg/kg body weight for sucralose to mitigate these risks.

## Author contributions

AS: Conceptualization, Formal analysis, Resources, Writing – original draft. SS: Conceptualization, Data curation, Visualization, Writing – original draft. RB: Data curation, Methodology, Project administration, Writing – review & editing. SV: Data curation, Resources, Visualization, Writing – original draft. CV: Formal analysis, Supervision, Validation, Writing – review & editing.
